# UniCoracle: automated hierarchical feature selection via bottom-up propagation and top-down skimming using the UniCorP algorithm and the Coracle machine-learning framework

**DOI:** 10.1093/bioinformatics/btag507

**Published:** 2026-07-09

**Authors:** Sebastian Staab, Anny Cardénas, Raquel S Peixoto, Falk Schreiber, Christian R Voolstra

**Affiliations:** Department of Biology, University of Konstanz, Konstanz 78457, Germany; Department of Biology, University of Konstanz, Konstanz 78457, Germany; Department of Biology, American University, Washington, DC, United States; Red Sea Research Center (RSRC), Biological and Environmental Sciences and Engineering Division (BESE), King Abdullah University of Science and Technology (KAUST), Thuwal, Saudi Arabia; Department of Computer and Information Science, University of Konstanz, Konstanz 78457, Germany; Faculty of Information Technology, Monash University, Clayton, Victoria 3800, Australia; Department of Biology, University of Konstanz, Konstanz 78457, Germany; Red Sea Research Center (RSRC), Biological and Environmental Sciences and Engineering Division (BESE), King Abdullah University of Science and Technology (KAUST), Thuwal, Saudi Arabia

## Abstract

**Summary:**

Identifying meaningful associations between microbial communities and measured physiological or environmental variables becomes increasingly complex and computationally demanding given the continuous growth of microbiome datasets. The Coracle machine learning (ML) framework was recently developed to address this issue by integrating multiple data transformations, feature selection techniques, and ML models to yield condensed lists of features that align to target variables of interest. Further, we recently developed the UniCorP feature aggregation algorithm to identify uniquely correlated features (UNICORNs) based on the UniCor metric that iteratively enrich each taxonomic level in an automated bottom-up approach. Here we present UniCoracle, a fully automated analytical framework that integrates UniCorP’s bottom-up propagation approach with a subsequent and newly developed top-down skimming (TDS) strategy, implemented with the Coracle ML framework. This combined approach leverages the inherent taxonomic structure of microbiome community data (e.g., ASVs derived from 16S rRNA gene amplicon sequencing data) to maintain predictive stability, reduce computational runtime, and identify biologically meaningful taxonomic associations. We compare the original, non-hierarchical Coracle with the TDS Coracle method and the UniCoracle approach. Evaluations across the tested datasets show that UniCoracle achieves competitive or improved predictive performance relative to both Coracle’s multi-step and the TDS-based Coracle implementations and demonstrate UniCoracle’s improvements in predictive accuracy over both methods. UniCoracle provides full control over feature set size and runtime, offering a streamlined and user-friendly framework for biological hypothesis generation. It identifies features (e.g., bacterial taxa) at the lowest (most specific) hierarchical level (e.g., ASV or species within a taxonomic hierarchy) that are associated with continuous target variables.

**Availability:**

UniCoracle is freely accessible via a dedicated web server at micportal.org. The source code is open source and available on GitHub at github.com/SebastianStaab/UniCoracle.git and Zenodo at https://doi.org/10.5281/zenodo.19050205.

## 1 Introduction

**Figure 1:**
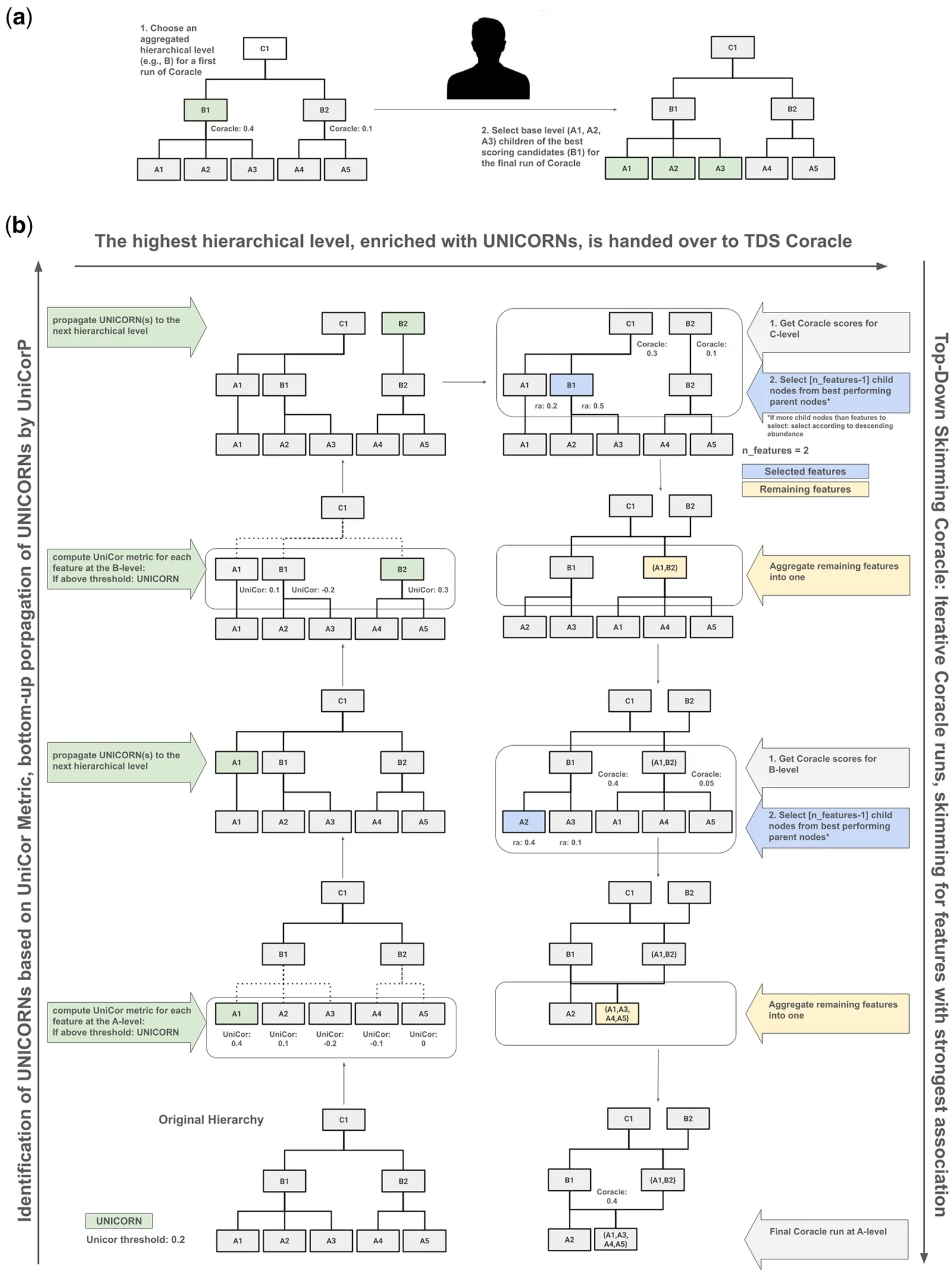
Coracle and UniCoracle workflow. (a) Recommended Coracle workflow. To obtain results at the lowest hierarchical level (e.g., species/ASVs/OTUs), it is recommended to first run Coracle at a higher taxonomic level (e.g., family/order level). The highest scoring families/orders from the initial run are then used to obtain a feature set at the lowest level by selecting species/ASVs/OTUs within the families/orders of interest in a second Coracle run. Since the number of features heavily impacts the runtime complexity, this two-step approach allows the execution of Coracle on a greatly reduced feature set to obtain a feature list at the lowest, most specific level. (b) Illustrated UniCoracle workflow. UniCoracle is an automated and integrative workflow combining a UniCor metric-based UniCorP bottom-up propagation of select features with a top-down skimming (TDS)-based Coracle framework. First, in a bottom-up approach, the UniCorP algorithm iteratively propagates the most unique and promising features to the next hierarchical level based on the UniCor metric. Once the highest level is reached and enriched with UNICORNs, the Coracle framework identifies the most important features at the highest hierarchical level. A user-selected number of child-features (*n*_features) of the best performing parent-features is then kept to run on the next lower hierarchical level. This top-down skimming (TDS) technique selects only the most relevant features moving down the hierarchical annotation, and it results in a significantly reduced but enriched feature set at the lowest, most specific hierarchical level. This procedure can reduce runtime complexity considerably, especially for large datasets, while reducing noise and improving predictive model performance. Green cells depict identified UNICORNs in UniCorPs bottom-up propagation step. During the TDS Coracle steps, blue cells mark newly selected child features to be included in the next Coracle run, while yellow cells show the aggregation of all other child-features to the remaining features group.

Given the vast complexity of microbial assemblages across ecosystems and host organisms (Galand *et al*. 2023, Voolstra *et al*. 2024), reducing microbiome datasets to features (i.e., bacterial taxa) that align with a target variable of interest is desirable. Such dimensionality reduction enables the identification of biologically meaningful patterns, facilitates hypothesis testing, and enhances the interpretability of microbiome–environment relationships. For instance, hierarchy aware feature selection and engineering methods exploit taxonomic structure to reduce dimensionality and improve prediction (e.g. SHSEL, HFE, TaxaHFE, TASSO) (Ristoski and Paulheim 2014, Wang and Zhao 2017, Oudah and Henschel 2018, Oliver *et al*. 2023). However, many methods are tailored to classification rather than regression, and the majority are not suited to analyses at the lowest, most specific taxonomic level (Ristoski and Paulheim 2014, Wang and Zhao 2017, Oudah and Henschel 2018, Oliver *et al*. 2023). We have recently developed the machine learning (ML) framework Coracle (Staab *et al*. 2024) to identify microbiome-trait associations. Coracle employs an ensemble approach that combines multiple different data transformations, feature selection methods, and machine learning models to improve robustness and confidence when analyzing sparse and wide datasets with typically small sample sizes (many features in comparison to the number of samples). The resulting complexity is especially vulnerable to datasets with a large number of features. In microbiome community analyses, the desired output often requires the most specific, i.e., lowest, taxonomic annotation (e.g., species level, Amplicon Sequence Variant [ASV] level, or Operational Taxonomic Unit [OTU] level). In contrast to aggregated features (e.g., on the taxonomic family/order level), the most detailed annotation (e.g., species/ASVs/OTUs) implies the largest number of features, with consequences for runtime requirements. Thus, to control the number of features when running Coracle, a multi-step approach is recommended in which the microbiome community data is first run at a higher taxonomic level (e.g., family or order level), and in a subsequent step, the top performing groups (families/orders) of the first step are used to filter to species/ASV/OTU level and reduce its feature set to a feasible size (Fig. 1a) (Staab *et al*. 2024). This approach requires a rather detailed understanding of Coracle and microbiome data parsing. Users must manually select the taxonomic level for the first run, introducing subjectivity and increasing the risk of selecting non-representative feature groups. Additionally, users need to determine how many top-performing groups to include in the second run. If compositionality of the dataset is required, unselected features can be aggregated and included in the second step. Depending on the hierarchical structure of the dataset, more than two steps might be required to effectively reduce the size of the feature set. These decisions need empirical assessment or educated guesswork for optimal performance, introducing a source of error and variability. Standardizing and automating this process would improve usability and increase consistency and comparability of results (Voolstra *et al*. 2025). Recent advancements in automated leveraging of the hierarchical structure of taxonomically annotated microbiome datasets offer promising improvements. For instance, UniCorP (Staab *et al*. 2025) is an algorithm that first selects features of interest (UNICORNs) based on their uniqueness (computed from feature-target correlation and in-group feature-feature correlation). In a second step, identified UNICORNs undergo propagation–the iterative carry-over of informative features from the lowest, most specific, to the highest, least specific, hierarchical level–in a bottom-up approach, enabling an effective reduction of feature set size while preserving critical information in the form of uniquely correlated features. Here we present UniCoracle, an algorithm that combines UniCorP’s bottom-up propagation approach with a subsequent top-down skimming (TDS) approach—the iterative filtering of child features based on parent-node performance—utilizing the Coracle ML framework. While the bottom-up UniCorP algorithm captures important features at the lowest, most specific annotation levels and then refines the feature set progressively, the subsequent top-down skimming approach introduced here allows us to return to the lowest hierarchical level for all feature annotations, reducing the noise of the dataset and providing the highest feature resolution. This two-tiered approach minimizes the risk of omitting key features while filtering out less informative ones. UniCoracle’s parameters can be tailored to match the size and characteristics of the desired output feature set. We compare three approaches: (a) the recommended multi-step approach for users running Coracle (Staab *et al*. 2024); (b) a TDS Coracle approach without UniCorP, and (c) UniCoracle that implements an approach combining UniCorP’s bottom up propagation of UNICORNs with the subsequent TDS top-down skimming procedure employing the Coracle ML framework. All analytical metrics are derived from Coracle’s result output. Using the example datasets provided with Coracle (Staab *et al*. 2024) and UniCor/UniCorP (Staab *et al*. 2025), we demonstrate competitive predictive performance, alongside improvements in runtime efficiency, automation, and error minimization within the evaluated datasets. Thus, we recommend UniCoracle for identifying features associated with a target variable in large datasets when the most detailed taxonomic resolution is required and noise and computational complexity are of concern.

## 2 Implementation—UniCoracle algorithm

UniCoracle builds on recent advancements in exploiting hierarchical structures to refine feature sets and identify key features. The workflow (Fig. 1b) integrates the bottom-up approach of UniCorP (Staab *et al*. 2025) with a top-down approach using Coracle’s scores to skim the feature set (TDS).

### 2.1 Bottom-up propagation: UniCorP

UniCorP is the bottom-up component of the UniCoracle workflow that enriches higher taxonomic levels with informative features from lower levels. The algorithm starts at the lowest (most specific) hierarchical level and propagates uniquely correlated features (UNICORNs) upward through the taxonomic hierarchy. At each taxonomic level, UniCorP computes the UniCor metric for each feature based on its correlation with the target variable minus its average correlation with other features in the same taxonomic group. Features exceeding a user-defined threshold or ranking among the top-k highest scores are identified as UNICORNs and propagated to the next higher level. This process repeats until the highest taxonomic level is reached and enriched with the most informative features. Scoring supports both Pearson’s or Spearman’s correlation and the optional data transformations include relative abundance normalization, centered log-ratio transformation, or using raw counts directly. These settings affect scoring only, while the propagation mechanism is unchanged. The UniCor metric and the UniCorP algorithm are explained in detail in previous work (Staab *et al*. 2025).

### 2.2 Top-down skimming: TDS

Once the top taxonomic level (Fig. 1b: C-level) has been enriched with UNICORNs, the Top-Down Skimming (TDS) approach is employed. Employing Coracle (Staab *et al*. 2024), the top scoring features at the highest level (Fig. 1b: C-level) will be determined to select child features at the next hierarchical level (B-level) for further analysis, up to a user-provided threshold (*n*_features). The *n*_features threshold specifies the maximum number of features to include at each hierarchical level. This ensures consistent feature set size, leading to predictable runtimes while avoiding issues with excessively large or small feature sets. We recommend keeping *n*_features within a few hundred features due to runtime limitations, with initial trials starting at the lower end. A minimum threshold of two features is necessary to keep compositionality. If the *n*_features threshold is reached before all child features (Fig. 1b: B-level) of one parent feature (Fig. 1b: C-level) can be included, selection is based on descending abundance. The remaining features are aggregated into one child feature called remaining features (Fig. 1b: B-level). This preserves compositionality, but high abundance of the remaining features might lead to a source of bias. The selected features, including the remaining features, are then re-evaluated using Coracle, iteratively repeating Coracle for all hierarchical levels (e.g., C-level to B-level, then B-level to A-level). This results in a focused feature set at the lowest, most specific hierarchical level, where Coracle is applied once more to identify the most promising features. By systematically reducing the feature space while preserving key information at each iteration, i.e. taxonomic level, runtime complexity is minimized and predictive performance enhanced. The Coracle ML framework is explained in detail in previous work (Staab *et al*. 2024).

UniCoracle is available as a Python implementation (github.com/SebastianStaab/UniCoracle.git) and as a web application at micportal.org. On the web server the configuration is streamlined: Selection uses top_*k* = *n*_features for predictable dimensionality and runtime, while the correlation metric and data transformation for UniCorP remain user-selectable. As recommended with UniCorP (Staab *et al*. 2025), we use Spearman correlation and relative_abundance transformation for microbiome community datasets. The UniCoracle workflow requires three input files (‘datasets’) (Table 1):

Algorithm Pseudocode–UniCoracle Input  X : ASV abundance table **(**samples × ASVs)     y : continuous target variable (samples)   T : taxonomic hierarchy (ASVs × levels)   n : per-level feature budget   uc : flag—apply UniCorP pre-filter**    θ**, k : UniCorP threshold/top-k (mutually exclusive*)*

Output  S : Coracle result at the lowest retained taxonomic level

function UniCoracle(X, y, T, n, uc, _**θ**_, k): 

# 1. Optional bottom-up UniCorP pre-filter of ASVs

  if uc:       X_f ← UniCorP(X, y, T, threshold =** θ**, top_k = k) # returns UniCor-filtered, taxonomy-annotated ASVs  else:

       X_f ← join(X, T)

# 2. Top-Down Skimming (TDS) with Coracle

for i in 0* .* |levels| − 1:

   X_i ← groupby_sum(X_f, levels[i]) # aggregate at current level

    S ← Coracle(X_i, y) # per-group importance-weighted scores

  if maxR^2^(S) ≤ 0:    break     # break if no model has an R^2^ > 0

  if i == |levels| − 1:   break    # break at the last level

  # Select children under the feature budget n

  selected ← []

  for taxon in sort_desc(S):

  if S[taxon] ≤ 0:    break    # break if taxon has score 0 (not selected in any model R^2^ > 0)

  if |selected| ≥ n:  break     # break if feature budget is reached

  children ← groupby_sum(X_f where levels[i] == taxon, levels[i + 1]) # aggregate at children level

   if |selected| + |children| ≤ n − 1:

     selected ← selected ∪ children

   else:

      m ← n − |selected| − 1    # reserve 1 slot selected ← selected ∪ top_m(children, by=abundance)

      break

    # Compositionality safeguard: bucket non-selected children

    if |selected| == n − 1:          label children of levels[i + 1] not in selected as “remaining features”

          selected ← selected ∪ {“remaining features”} # Propagate to next level

          X_f ← X_f where levels[i + 1] ∈ selected return *S*

Example files [1. x (ASV level), 2. y (ED50), 3. hierarchy (taxonomic)] are available as Supplementary Files [S1-S3], available as supplementary data at *Bioinformatics* online or at micportal.org.

UniCoracle produces the same* .*csv output file format as Coracle, i.e., a ranked list of features sorted by an ensemble score that integrates predictive performance, importance, and robustness. Reported key metrics include regression coefficients that denote the direction of association, model-specific validation statistics such as *R*^2^ and mean squared error (MSE), and detailed feature importance sub-measures for each analytical path in the pipeline.

## 3 Application/Results

### 3.1 *Stylophora pistillata* coral heat stress microbiome dataset

We tested the performance of all three approaches (multi-step Coracle, TDS Coracle, and UniCoracle) on a dataset consisting of 28 samples of the coral *Stylophora* *pistillata* (Voolstra *et al*. 2021), their standardized thermal tolerance thresholds (ED50s), and their bacterial composition (16S rRNA gene amplicon ASVs annotated in their taxonomic hierarchy). This dataset was used to test the original Coracle implementation (Staab *et al*. 2024), allowing for a direct comparison with TDS Coracle and UniCoracle. Since Coracle’s runtime complexity scales polynomially with the size of the feature set, Coracle is recommended to be run in a multi-step approach, i.e., at multiple taxonomic levels, starting with a higher taxonomic level to generate a select feature set for processing at a lower taxonomic level (Staab *et al*. 2024). The recommended level for the initial Coracle run is family as it provides a balance between specificity and feature set complexity (Staab *et al*. 2024). In the original study (Staab *et al*. 2024), the ASVs of the 10 best performing families were then used for the second step, drastically reducing the number of features (355 ASVs) compared to the unfiltered dataset size (4,568 ASVs). In comparison to Coracle, the TDS Coracle approach and UniCoracle workflow both provide full control over the number of features included in the model, allowing precise regulation of the runtime. Also, the compositionality of the dataset is retained automatically, while this task requires manual aggregation for the remaining features for each Coracle run when using a mutli-step Coracle approach. In the UniCoracle workflow, starting the UniCorP bottom-up approach at the lowest taxonomic level of the original dataset, enriches the highest hierarchical level with UNICORNs. These UNICORNs have no extra children nodes beyond themselves, decreasing the number of expected children nodes per parent node, once the Coracle-based TDS step of the UniCoracle workflow is initiated. For example, if a previously propagated UNICORN is selected in the subsequent TDS process, it will not be split up into multiple children nodes at the next lower level, but is retained as a single feature. Conversely, if the original parent node of a UNICORN is chosen, it generates one fewer child node, as the UNICORN is no longer considered part of its original child group. This approach allows a broader, more controlled selection of putative important features. Within the datasets evaluated here, UniCoracle demonstrates competitive predictive performance and reduced computational runtimes compared to a multi-step Coracle approach. Additionally, the automation of the process enhances usability, thereby expanding the user base, while reducing the risk of unintended user errors.

While all three approaches achieve good predictive performances at the ASV level, UniCoracle and TDS Coracle achieve this with a significantly more focused feature set while still slightly outperforming the two-step Coracle approach (Table 2). Prevalent within the top scoring features is the genus *Endozoicomonas* in the TDS Coracle and UniCoracle approaches (ASV0003 and ASV0011 in TDS Coracle and UniCoracle; additionally, ASV0005 in TDS Coracle) (Supplementary Files S5.1-S5.3, available as supplementary data at *Bioinformatics* online). This suggests that the two-step Coracle approach may overlook important features, since bacteria of the genus *Endozoicomonas* are common, prevalent, and functionally important members of the coral microbiome (Neave *et al*. 2016, 2017, Pogoreutz *et al*. 2022). Additional high scores were further obtained by the genera *Alteromonas* (ASV0004 in TDS Coracle, ASV0017, ASV0019, and ASV0021 in two-step Coracle), *Rhodoluna* (ASV0101 in two-step Coracle), *Nonlabens* (ASV0013 in UniCoracle), and *Donghicola* (ASV0032 in UniCoracle). While *Alteromonas* and *Endozoicomonas* are typical coral microbiome associates (Voolstra *et al*. 2024), *Rhodoluna*, *Nonlabens*, and *Donghicola* may provide novel target genera for assessment of contribution to coral thermal tolerance.

**Table 1:** Dimensions of input files.

Input file	Dimensions: row*columns
Quantitative feature set	n*m
Hierarchy	m*l
Continuous target variable	n*1

*n* = number of samples, *m* = number of features, l = number of hierarchical levels. Three types of files are needed: (1) Quantitative feature set file, e.g., a microbial community data matrix with rows as samples and columns as ASVs; (2) Hierarchy file, e.g., taxonomic ranks for ASVs (Phylum, Class, Order, Family, Genus, and Species); (3) Continuous target variable file, e.g., ED50 thermal tolerances for each sample. All files can be* .*csv or* .*tsv format. Example input files are available at micportal.org or in the Supplementary Files S1–S3, available as supplementary data at *Bioinformatics* online.

**Table 2:** Performance comparison of three approaches (two-step Coracle, TDS Coracle, UniCoracle) to identify features associated with a target variable of interest (ED50 standardized thermal tolerances) based on the *Stylophora pistillata* coral heat stress microbiome dataset (Voolstra *et al.* 2021, Staab *et al.* 2024, 2025).

Method	#features	Avg *R*_2_
Two-step Coracle (ASV level)	355	0.5375
TDS Coracle (ASV level)	150	0.5608
UniCoracle (ASV level)	150	0.5827

The table reports the average *R*^2^ value across all models and the number of features (in the case of TDS Coracle and UniCoracle, results for *n*_features = 150 are shown as a balance between runtime and predictive performance; a full comparison of different *n*_features parameter choices is in Supplementary File S4, available as supplementary data at *Bioinformatics* online).

**Table 3:** Number of samples and number of bacterial ASVs (features) for the four most common coral hosts (genus level) of the CDIV *Tara* Pacific dataset.

Coral Host	*Acropora*	*Montipora*	*Porites*	*Pocillopora*
# samples	220	121	100	82
# bacterial ASVs	48,877	96,520	27,792	50,824

### 3.2 *Tara* Pacific Coral Diversity (CDIV) dataset

The *Tara* Pacific CDIV dataset comprises 16S rRNA gene amplicon microbiome assemblage data of 2,470 coral specimens sampled across the Pacific Ocean (Bourdin *et al.* 2022, Hume *et al*. 2022, Ruscheweyh *et al*. 2022) under the *Tara* Pacific expedition (Planes *et al*. 2019), contextualized to a large array of environmental variables (Lombard *et al*. 2023). We applied all three approaches to the four most abundant coral genera (*Acropora*, *Montipora*, *Pocillopora*, *Porites*) sampled. The dataset, prepared as described previously (Staab *et al*. 2025), entails bacterial abundance data from corals (Ruscheweyh *et al*. 2022) matched with the taxonomic annotation at genus-level for the coral host (Hume *et al*. 2022) and the Sea Surface Temperature (SST) at each sampling station as the target variable of interest (Bourdin *et al.* 2022) (Table 3).

Due to the dataset size (on average over 50, 000 ASVs for each coral genus), a two-step approach is computationally unfeasible, thus we implement a three-step Coracle approach at the order, genus, and ASV levels. To maintain a manageable number of features, only a few of the top-scoring feature groups were manually selected at each step.The three-step Coracle approach required two steps of manual feature selection (Order -> Genus, Genus -> ASV) in order to become computationally feasible, leading to varying feature set sizes and performances. In contrast, TDS Coracle and UniCoracle produced stable and significantly reduced feature set sizes, set by the user’s preference. While average performance across coral genera (Table 4) in the Coracle framework (Staab *et al*. 2024) varies between the three-step Coracle and the TDS Coracle approaches, UniCoracle shows substantial improvements, yielding robust results across all four coral genera. This highlights the added value of incorporating the UniCor metric and UniCorP algorithm with the TDS algorithm in UniCoracle compared to a TDS Coracle only approach. For *Acropora*, UniCoracle scored ASV13 (genus *Prochlorococcus* MIT9313), ASV30 (genus *Endozoicomonas*), ASV262 (genus *Pseudoalteromonas*), and ASV17 (genus *Catenococcus*) the highest. By comparison, for the three-step Coracle approach and the TDS Coracle approaches, ASV13 was also the highest scoring feature. Additionally, the three-step Coracle approach gave high scores for ASV17 and ASV4 (genus *Catenococcus*). ASV16 (genus *Prochlorococcus* MIT9313) and ASV59 (genus *Prochlorococcus* MIT9313) scored highly in the three-step Coracle approach and the TDS Coracle approaches, while also receiving modestly good scores from UniCoracle. For *Montipora*, UniCoracle identified ASV308 (genus *Pseudoalteromonas*), ASV27597, ASV 15112, and ASV16771 (all genus *Rubritalea*) as the highest scoring features. By comparison, the three-step Coracle and TDS Coracle approaches shared ASV3066, ASV16771, ASV958, ASV6814, and ASV12121 (all genus *Rubritalea*) in their top feature scores, some of which were absent in the UniCoracle final feature set. For *Porites*, UniCoracle identified ASV3066 (genus *Rubritalea*), ASV308 (genus *Pseudoalteromonas*), ASV663 (genus *Endozoicomonas*), and ASV2542 (genus *Endozoicomonas*) among the top-scoring features. For *Pocillopora*, the top scoring features using UniCoracle were ASV1600, ASV30, ASV567 (all genus *Endozoicomonas*), and ASV104 (genus *Halomonas*). The TDS Coracle approach identified ASV578, ASV13 (both genus *Prochlorococcus* MIT9313), ASV262 (genus *Pseudoalteromonas*), and ASV308 (genus *Pseudoalteromonas*), while the top-scoring feature for the three-step Coracle approach was ASV1250 (genus KI89A clade). Result files for the three approaches are available as Supplement (S5.1–S5.15), available as supplementary data at *Bioinformatics* online.

**Table 4:** Performance comparison of three approaches (three-step Coracle, TDS Coracle, UniCoracle) to identify features associated with a target variable of interest (SST) based on the *Tara* Pacific Coral Diversity (CDIV) dataset.

	# Features (ASVs)				Avg. *R*_2_			
Method	*Acropora*	*Montipora*	*Porites*	*Pocillopora*	*Acropora*	*Montipora*	*Porites*	*Pocilopora*
Three-step Coracle	427	329	223	370	0.3395	0.1781	0	0.1319
TDS Coracle	150	150	150	150	0.3659	0.0438	0	0.276
UniCoracle	150	150	150	150	0.6092	0.2146	0.3529	0.5578

The number of features (i.e., ASVs) is used as a proxy for runtime and is displayed only for the final Coracle step. For TDS Coracle and UniCoracle, the *n*_features parameter is set to 150, controlling the effective size of the feature set and thus the runtime. The genus *Porites* received zero scores for the three-step Coracle and TDS Coracle workflows, resulting in the TDS Coracle algorithm not being able to select top scoring groups.

**Table 5:** Comparison of automation, data handling, compositional integrity, runtime complexity, diversity, and predictive performance of feature selection for Coracle, TDS Coracle, and UniCoracle.

	Original Coracle Approach	Top-Down Skimming (TDS) Coracle Approach	UniCoracle workflow (combining UniCorP and TDS Coracle)
Automation	May require multiple steps at different hierarchical levels for larger feature sets	Iterates automatically through all hierarchical levels top-down	Iterates automatically through all hierarchical levels bottom-up and top-down
Manual data handling	Requires manual effort to handle the data in between those steps	No manual data handling after the preparation of the initial dataset	No manual data handling after the preparation of the initial dataset
Compositionality	Not automatically ensured	Automatically ensured	Automatically ensured
Runtime complexity	Less control over the size of the feature set and runtime complexity	Full control over the size of the feature set and runtime complexity	Full control over the size of the feature set and runtime complexity
Feature selection	Risk of omitting unique, important features	Risk of omitting unique, important features	Unique features are separated from noise, leading to diverse feature sets and stable performances

**Figure 2:**
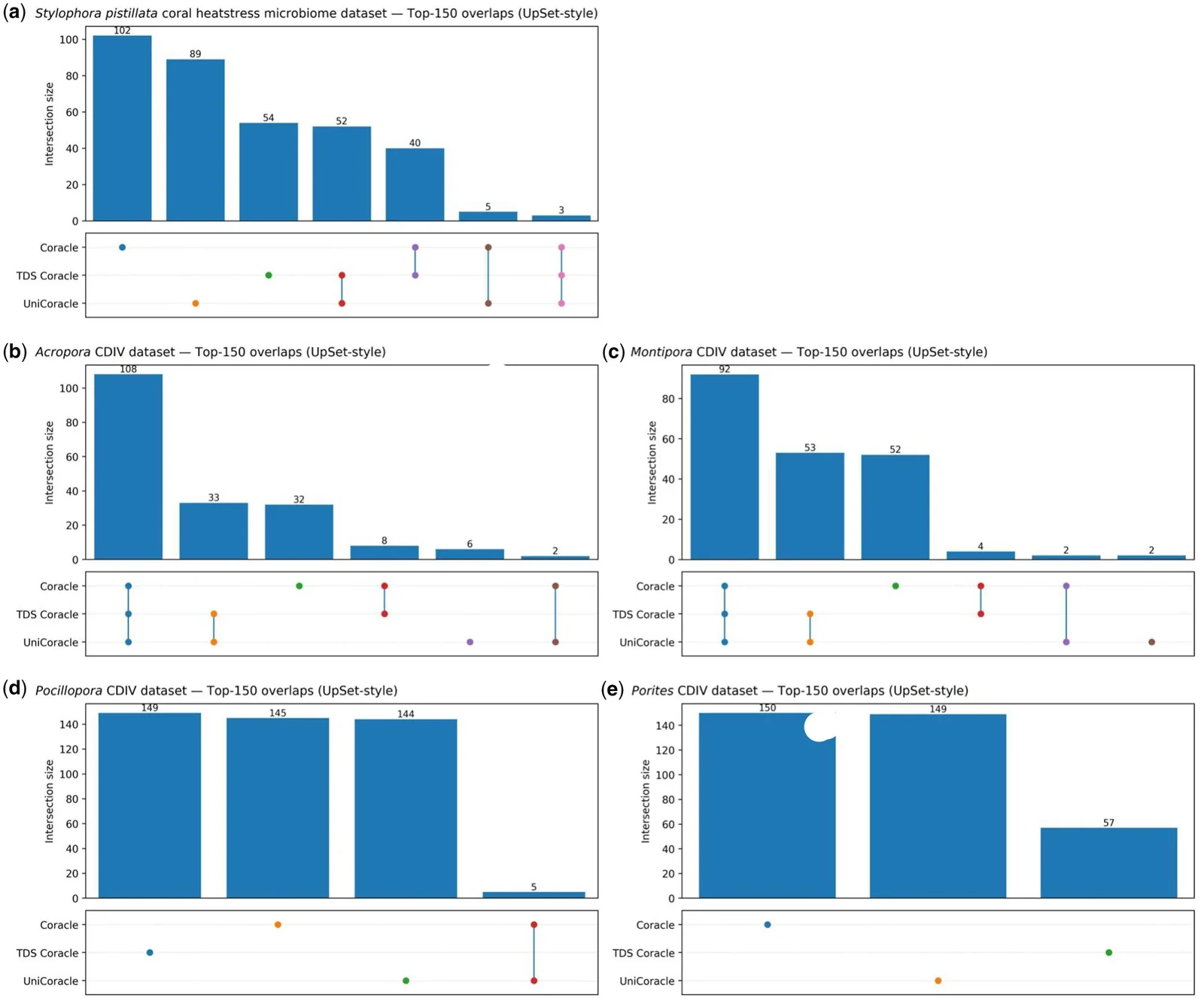
Overlap of different feature selection approaches (Coracle, TDS Coracle, UniCoracle). Depicted are UpSet analyses of the top-150 features (manually selected for Coracle, automatically selected for TDS Coracle and UniCoracle using *n*_features = 150). The comparison reveals coral species-/genus-specific results with variable correspondence between analyses. (a) The *Stylophora pistillata* coral heat stress microbiome dataset shows modest overlap and only small performance differences. (b)–(e) For the *Tara* Pacific Coral Diversity (CDIV) dataset for the genus *Acropora* (b), the methods overlap widely, and UniCoracle contributes a small set of unique features that aligns with clear performance gains (Table 3). The genus *Montipora* (c) displays broad overlap with similar performance across methods. The genus *Pocillopora* (d) exhibits minimal overlap, and the performance gap is larger and UniCoracle outperforms other methods (Table 3). For the genus *Porites*, multi-step Coracle and TDS Coracle produce non-informative fits (*R*^2^ = 0), while UniCoracle yields meaningful predictions (Table 3).

We note the lack of overlap in identified features (i.e., ASVs) between coral genera (Supplementary Files S5.1–S5.15), available as supplementary data at *Bioinformatics* online (Figure 2), supporting the notion that coral microbiomes are species-specific (Hochart *et al*. 2023, Voolstra *et al*. 2024).

## 4 Discussion

In this study, we devised UniCoracle, a fully automated analytical framework that combines UniCorP’s bottom-up propagation approach with a subsequent top-down skimming (TDS) approach employing the Coracle ML framework. To assess UniCoracle’s performance, we compared multi-step Coracle, TDS Coracle, and UniCoracle across a small and across a large microbiome dataset to characterize performance and runtime gains from hierarchical feature aggregation (UniCorP) and Top-Down Skimming (TDS). Since the UniCoracle workflow builds upon Coracle, it remains equally vulnerable to small sample sizes, which is often characteristic of microbiome datasets. Although the sample size is given by the dataset, the UniCoracle approach is expected to reduce dataset noise, potentially enhancing performance on smaller datasets.

To evaluate model robustness and sensitivity to sample size reduction, we utilized a jackknifing approach (*n*_features = 10). To do this, we systematically removed subsets of samples to scale the retained dataset from 27 down to 5 samples (10 subsets for each sample-set size). Following this, the model maintained a stable mean *R*^2^ between 0.43 and 0.53 when trained on at least 20 samples. Below this threshold, the variance of predictive performance increased substantially. The models displayed a mean *R*^2^ between 0.30 and 0.53 on sample sizes between 10 and 19, and they ultimately reached a mean *R*^2^ of 0.10 at 5 retained samples (Supplementary File S7, available as supplementary data at *Bioinformatics* online), supporting the findings of a similar analysis on Coracle, i.e., that a minimal sample size of 20 is recommended when running both Coracle and UniCoracle (Staab *et al*. 2024).

Although UniCoracle demonstrates stable predictive performance on the evaluated datasets, performing global feature selection prior to cross-validation introduces partial information leakage. Nested cross-validation on every level of the hierarchy remains unfeasible due to a computational complexity scaling by a factor of n*l, where *n* is the number of samples and l represents the number of hierarchical levels. To validate statistical significance despite this partial leakage, we performed a permutation test (*n*_features = 10 to manage computational demands) on the *Stylophora pistillata* dataset (28 samples, 4,568 ASVs). We randomly shuffled the target variable (i.e., ED50) 100 times prior to model training. The original model achieved an *R*^2^ of 0.50, with most permuted models yielding no meaningful results at all (*R*^2^ ≤ 0) and none exceeding the original model, resulting in a *P* value of 0.01. This supports the notion that the predictive performance captures a robust biological signal rather than an artifact of random chance (Supplementary Files S6.1–S6.2, available as supplementary data at *Bioinformatics* online).

Aggregating unselected child features into a “remaining features” group maintains compositional integrity but may introduce model-specific biases. For instance, Random Forests potentially exhibit a selection bias toward dominant aggregate features because their higher variance provides the algorithm with more potential split points to minimize error, often inflating importance scores for uninformative noise (Strobl *et al*. 2007). Our assessment of the results confirms this effect: the “remaining features” group consistently scores above most individual features, yet rarely reaches the very top ranks (Supplementary Files S5.3, S5.6, S5.9, S5.12, S5.15, available as supplementary data at *Bioinformatics* online), supporting the presence of some form of selection bias for highly abundant features. However, the presence of specific features outranking the aggregate group demonstrates that individual features possess a stronger, more unique association with the target variable than the rest of the community combined. Thus, we have chosen to mitigate this effect by an implemented tie-breaking heuristic that prioritizes the aggregation of features with lower relative abundance when importance scores are identical.

The choice of parameters (UniCor parameters and *n*_features) significantly impacts the predictive performance and runtime of UniCoracle. Limiting the number of features at each level (*n*_features) helps control noise, computational complexity, and runtime. However, setting *n*_features too low may exclude relevant features, while setting *n*_features too high can introduce noise and significantly increases runtime. We recommend starting with *n*_features = 100 to ensure a diverse feature representation, while maintaining computationally manageable runtimes (“out-of-the-box” runtime on a commercially available laptop equipped with an AMD Ryzen 55 500U, i.e., 6 cores at 2.1 GHz, and 8GB of RAM is 25 min and 14 min for the *Stylophora pistillata* dataset with *n*_features = 100 and *n*_features = 50, respectively).

For the initial bottom-up selection of features in UniCoracle, the UniCor selection determines which features are propagated through the hierarchical levels. The selection rule at each level can be set as top-*k* or as a threshold on the UniCor score. The top-*k* option propagates the *k* highest-scoring features (UNICORNs) and keeps dimensionality and runtime predictable. It also aligns naturally with the top-down skimming stage, where *n*_features limits the number of children carried forward, so we recommend top_*k* = *n*_features and set this as the default. Thresholding retains only groups whose UniCor score exceeds a chosen value. Scores lie in the interval -0.5 to 1 and increasing the threshold reduces the number of propagated features. Very high values can prevent propagation at all. Users can also choose the scoring configuration: The correlation metric can be Pearson’s or Spearman’s, and the optional preprocessing step can apply relative abundance or CLR before scoring to reflect compositional structure. These settings affect how scores are computed and should be set according to the characteristics of the dataset and the goals of the analysis.

Using two different datasets, a comparatively small (28 samples, 4,568 ASVs) and a rather large (523 samples across 4 coral genera with on average over 50,000 ASVs) we show the performance difference of Coracle, TDS Coracle, and UniCoracle. TDS Coracle alone shows variable performance improvements relative to the multi-step Coracle approach, whereas UniCoracle consistently achieves competitive or improved predictive performance across the evaluated datasets. This advantage is particularly evident in the larger dataset, where substantial performance gains are observed across several coral genera. Additionally, UniCoracles runtime is substantially lower than that of the multi-step Coracle approach and remains comparable to TDS Coracle, as the UniCorP step adds only marginal computational complexity, even for medium- or smaller-sized datasets. Ultimately, we recommend using UniCoracle over the multi-step Coracle approach and TDS Coracle due to its greater control over runtime, automation, and more robust predictive performance. Together, these characteristics position UniCoracle as a highly effective framework for identifying biologically relevant candidate taxa, providing a streamlined and reliable basis for downstream hypothesis validation (Table 5).

To select target features that are correlated to a target variable of interest, the UniCoracle workflow introduces several key improvements over the original Coracle framework (Staab *et al*. 2024). These improvements include automated feature propagation, noise reduction, optimized runtime, and enhanced ease of use (Table 4), eliminating the need for manual user intervention once UniCorP and *n*_features parameters are chosen. By leveraging inherent hierarchical structures through a combination of the UniCor metric in conjunction with the UniCorP’s bottom-up feature propagation and the top-down skimming (TDS) algorithm using Coracle, the UniCoracle workflow efficiently reduces computational complexity while preserving and selecting relevant features. Although predictive performance is an important metric for model evaluation, UniCoracle’s primary objective remains the identification of meaningful, interpretable relationships in continuous data to serve as a foundation for downstream biological hypotheses. UniCoracle can be applied to datasets of comprising continous variables with tree-like, hierarchically structured features. The UniCoracle workflow is broadly applicable to hierarchical datasets across diverse disciplines, such as microbiomics and genomics, offering increased computational efficiency as well as convenience through its automated processes in selecting features correlated to a target variable of interest. The code for UniCoracle, UniCor/UniCorP, and Coracle can be openly accessible via github.com/SebastianStaab/UniCoracle.git, the fully functional UniCoracle workflow, with UniCorP integration, is available at micportal.org, along with tutorials and example data. We invite the community to further explore its potential and apply it to diverse problems to unlock new insights in microbiome research and beyond.

## Supplementary Material

btag507_Supplementary_Data

## Data Availability

The data underlying this article are available in the article and in its online supplementary material.
